# Hikikomori as a culturally situated expression of neurodivergence: A two‐stage international analysis

**DOI:** 10.1002/pcn5.70358

**Published:** 2026-06-17

**Authors:** Emily Ravenhill, William Farr

**Affiliations:** ^1^ Faculty of Education University of Cambridge Cambridge UK

**Keywords:** cultural manifestation, hikikomori, neurodivergence, neurodiversity

## Abstract

**Aim:**

Hikikomori is a behavior characterized by prolonged social withdrawal, identified in 1990s Japan, and with increasing global recognition. Over two decades of research, the phenomenon remains clinically ambiguous; debate persists over whether hikikomori represents a psychiatric disorder, a behavioral response, or an adaptive coping strategy. This review examines the intersection between hikikomori and neurodivergent traits, focusing on autism spectrum characteristics, and ecological and cultural contexts that shape presentation.

**Methods:**

Two‐stage literature review. Stage 1 comprised a scoping review of diagnostic criteria for autism and behavioral features of hikikomori, identifying overlapping social communication differences, sensory sensitivities, and restricted interests. Stage 2 employed a targeted Preferred Reporting Items for Systematic reviews and Meta‐Analyses extension for Scoping Reviews (PRISMA‐ScR) approach, synthesizing international literature across quantitative, qualitative, and mixed‐method studies. Findings followed three research questions: overlap between autism and hikikomori; Japanese environmental and societal triggers; and whether hikikomori can be understood as an adaptive response.

**Results:**

Results indicate nuanced convergence between autistic traits and hikikomori behaviors. Social communication difficulties, engagement in repetitive activities, and sensory sensitivities appear in both populations. Developmental trajectories differ; hikikomori emerge in adolescence/early adulthood. Environmental pressures, including academic expectations, family dynamics, and cultural norms around conformity, exacerbate withdrawal behaviors. Adaptive perspectives suggest that withdrawal is a protective mechanism, facilitating sensory regulation, psychological coping, and engagement in preferred activities. Pathological outcomes arise when isolation is prolonged/unsupported.

**Conclusion:**

This review underscores important ecological and neurodiversity‐informed frameworks in understanding hikikomori. Implications for practice include flexible support strategies, culturally sensitive assessment, and recognizing potential adaptive functions of withdrawal. Future research should be longitudinal, first‐person experiences, and interaction between neurodivergence and environmental stressors across cultures.

## Graphical abstract

Hikikomori, a behavioral pattern of prolonged social withdrawal first identified in Japan in the 1990s, overlaps with neurodivergent traits, particularly autism, and is shaped by ecological and cultural contexts. Our two‐stage literature review highlights converging social communication differences, sensory sensitivities, and repetitive behaviors, whilst environmental pressures and cultural expectations exacerbate withdrawal, suggesting both adaptive and pathological outcomes. Findings indicate the need for culturally sensitive assessment and flexible support strategies that recognize potential protective functions of withdrawal.

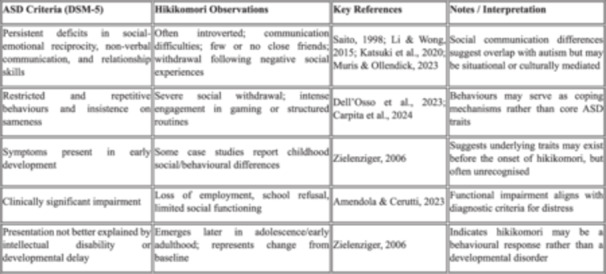

## INTRODUCTION

### Background

Hikikomori (引き篭も)—defined as prolonged and extreme social withdrawal—predominantly affects adolescents and young adults and is typically characterized by a retreat from societal participation for 6 months or more.^1^ First documented in Japan,^2^ hikikomori has since been observed internationally, suggesting that it may represent a broader behavioral and social phenomenon rather than a country‐specific pathology.^3^, ^4^ Despite increasing recognition, the concept remains contentious. Researchers debate whether hikikomori constitutes a psychiatric disorder, a symptom of underlying mental health conditions, a cultural response to social pressure, or an adaptive coping strategy in response to overwhelming environmental demands.^5^, ^6^

Emerging research highlights the potential intersection between hikikomori and neurodivergent traits, particularly characteristics associated with autism spectrum conditions (ASC). Autism is clinically defined by persistent deficits in social communication and interaction, alongside restricted and repetitive patterns of behavior and sensory sensitivities.^7^ Hikikomori behaviors—including social withdrawal, an aversion to verbal communication, and a preference for engagement in highly structured, solitary activities—bear notable similarities to these core autistic traits.^8^, ^9^ However, unlike autism, which is developmental in nature and has traditionally been recognized from early childhood, hikikomori commonly emerges in adolescence or early adulthood, often following exposure to new environmental stressors.^10^

### Conceptual ambiguity and research rationale

The literature on hikikomori is characterized by conceptual ambiguity. Some scholars frame it as a psychiatric disorder, emphasizing comorbidities with depression, anxiety, and Internet Gaming Disorder.^11^, ^12^ Others perceive it as a culturally mediated behavioral response to rigid social systems, with adaptive or protective functions.^13^ A central challenge is distinguishing between pathology and adaptation, particularly for individuals with neurodivergent traits who may withdraw as a rational response to overwhelming social expectations or sensory overload.

Studies from Japan highlight systemic and cultural factors that can exacerbate vulnerability to withdrawal. Family expectations, educational pressure, bullying, and high‐context communication norms create a cultural landscape where young people with autistic traits may struggle to meet societal standards, precipitating withdrawal.^14^, ^15^ Conversely, international studies indicate that similar withdrawal behaviors can occur in other cultural contexts, influenced by socioeconomic instability, technological change, and shifting employment landscapes.^4^, ^16^

In this project, a two‐stage review was undertaken to integrate the international evidence base and clarify three research questions:

1. What evidence exists for an overlap between autism spectrum traits and hikikomori behaviors?
2. What environmental and societal factors contribute to hikikomori, particularly in Japan?
3. To what extent can hikikomori be conceptualized as an adaptive response rather than a pathological condition?

### Aims and contribution

This review aims to advance understanding of hikikomori by situating it within ecological and neurodiversity‐informed frameworks. It synthesizes international literature to:

− Examine behavioral and developmental overlaps with autism.
− Identify environmental and societal stressors that influence withdrawal.
− Explore the adaptive potential of social withdrawal, balancing considerations of pathology and functionality.

By combining scoping and systematic review methods, the study provides a comprehensive, integrative account that bridges psychiatric, psychosocial, and cross‐cultural perspectives, offering implications for practice, policy, and future research.

## THEORETICAL FRAMEWORK

### Ecological systems theory

Bronfenbrenner's ecological systems theory (1979) provides a comprehensive framework for understanding how individual behavior is shaped by nested environmental influences. The theory distinguishes between multiple levels:

− Microsystem: immediate environments such as family, school, and peers.
− Mesosystem: interactions between microsystems, for example, family–school communication.
− Exosystem: broader social systems with indirect influence, including policy and institutional structures.
− Macrosystem: cultural norms, societal values, and dominant narratives.
− Chronosystem: developmental transitions and historical or technological changes over time.

Applying this framework to hikikomori allows for an analysis that integrates individual neurodivergence with social, cultural, and systemic influences. For example, the microsystem highlights family dynamics and peer relationships, while the macrosystem contextualizes pressures related to social conformity and high‐stakes educational expectations in Japan.

### Neurodiversity perspective

The neurodiversity paradigm frames neurological differences, including autism, as variations in human cognition rather than inherently pathological conditions.^17^ Within this perspective, behaviors typically classified as maladaptive may serve functional or adaptive purposes in specific environments. For hikikomori, this lens emphasizes the potential for social withdrawal to act as a coping strategy, regulating sensory input, mitigating social anxiety, or facilitating engagement in preferred activities.

Combining ecological systems theory with neurodiversity provides a robust framework for interpreting hikikomori: it recognizes the interplay between individual traits and contextual demands while moving beyond purely medicalized or deficit‐based explanations. This approach is particularly useful in cross‐cultural contexts, where social norms, educational systems, and familial expectations may mediate the expression and consequences of withdrawal behaviors.

## METHODS

### Two‐stage review design

A two‐stage literature review was conducted to synthesize evidence on hikikomori and its intersection with autistic traits. Stage 1 involved a scoping review to map the broad landscape of existing research and identify preliminary themes. Stage 2 employed a focused review using Preferred Reporting Items for Systematic reviews and Meta‐Analyses extension for Scoping Reviews (PRISMA‐ScR) guidelines to critically appraise and synthesize selected studies.

### Stage 1: Scoping review

The scoping review followed Arksey and O'Malley's^18^ framework. Key search terms included *hikikomori*, *social withdrawal*, *autism*, *neurodivergence*, and *adaptive behavior*. Databases searched included PsycINFO, PubMed, Web of Science, and Google Scholar. A flexible, iterative search strategy was employed in keeping with Arksey and O'Malley's^18^ framework, which does not prescribe fixed search strings. No date restrictions were applied at this stage. Inclusion criteria were peer‐reviewed articles in English, studies involving adolescents or emerging adults, and publications addressing behavioral characteristics or developmental trajectories of hikikomori. Exclusion criteria were studies solely focused on pharmacological interventions or unrelated psychiatric conditions. Literature was restricted to English‐language publications to avoid inaccuracies arising from translation; this is acknowledged as a limitation of the review. Screening was conducted by the primary author, which is also acknowledged as a limitation of the review. Screening was conducted by the primary author, which is also acknowledged as a limitation. No formal disagreement resolution procedure was applicable given the single‐reviewer design.

Data extraction captured study characteristics (year, country, and population), methodology, key findings, and relevance to research questions. Thematic analysis identified recurrent patterns, including social communication differences, sensory sensitivities, and restricted or repetitive behaviors. This stage provided a broad understanding of potential overlaps between hikikomori and autistic traits and informed the design of Stage 2.

### Stage 2: PRISMA‐ScR review

The second stage adopted a PRISMA‐ScR approach to systematically collate evidence addressing the three research questions. Two databases were searched: PsycINFO and PubMed. The search was conducted in 2025, covering literature published between 2022 and 2025. The following search strings were used:

PubMed: (“hikikomori” OR “social withdrawal” OR “prolonged social isolation” OR “autism” OR “ASD” OR “autistic”) AND (“adaptive” OR “response” OR “coping strategy” OR “resilience” or “non‐pathological” OR “maladaptive” OR “psychosocial adaptation” OR “social functioning”) AND (“covid” OR “pandemic” OR “lockdown”).

PsycINFO: Hikikomori AND (“adaptive” OR “pathological” OR “response” OR “coping” OR “resilience” OR “maladaptive” OR “environmental” OR “covid” OR “pandemic” OR “lockdown”).

The search strategy replicated Stage 1 but applied stricter inclusion criteria: studies needed to examine either behavioral features of hikikomori, neurodivergent traits, or environmental/contextual influences, and report quantitative or qualitative findings. Literature was restricted to English‐language publications. Screening was conducted by the primary author using a two‐step process: initial screening of titles and abstracts, followed by full‐text review for unclear cases. The single‐reviewer approach is acknowledged as a limitation. Given the heterogeneity of study designs included in the synthesis, studies were categorized according to evidential level to aid interpretation. Meta‐analyses, systematic reviews, and longitudinal studies were considered to provide the strongest evidence base. Cross‐sectional surveys, clinical interview studies, and scale development studies were classified as moderate evidence. Narrative reviews and case studies were treated as exploratory and interpreted with appropriate caution. Conclusions are weighted throughout the synthesis in line with these distinctions.

**Figure 1:**
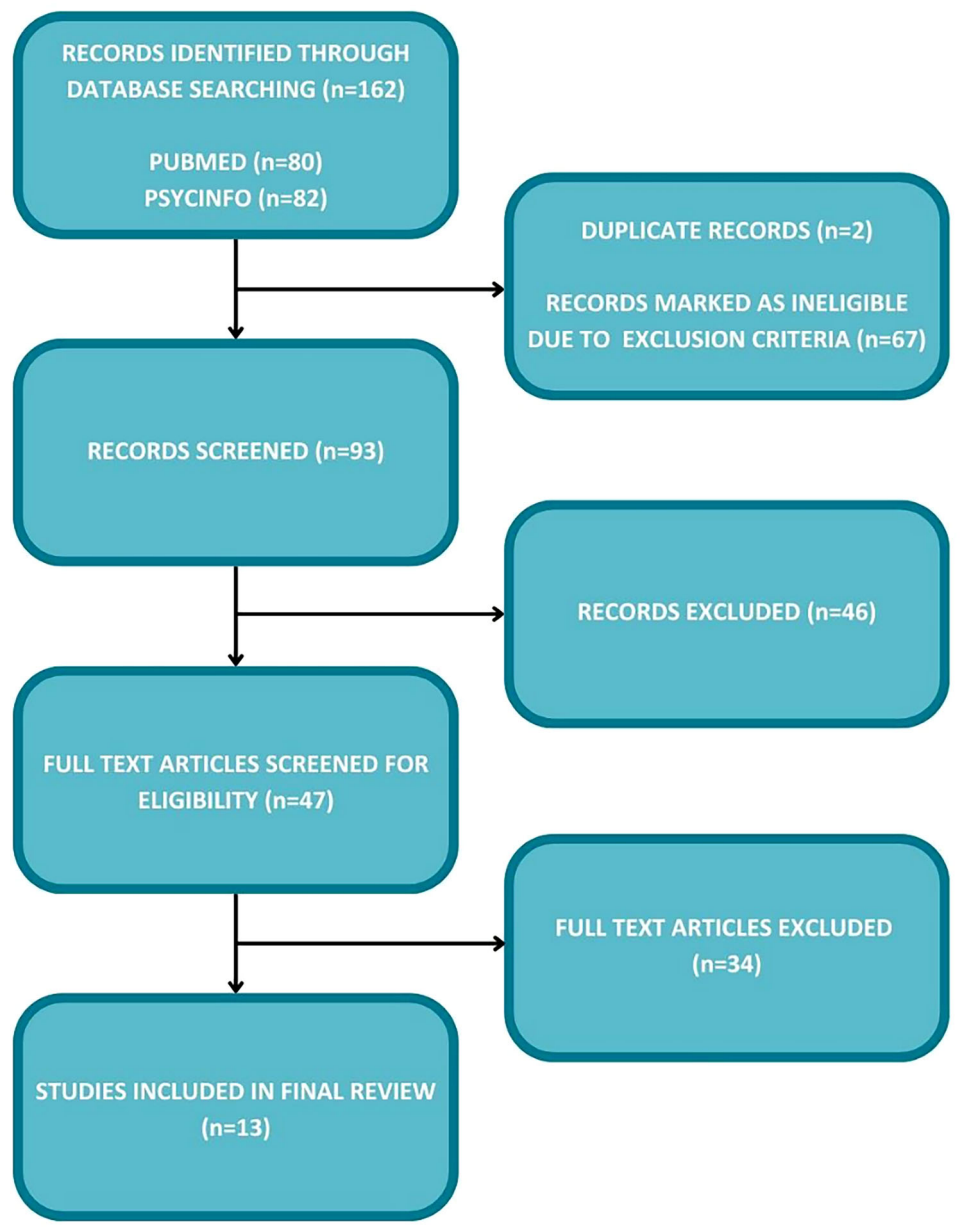
Preferred Reporting Items for Systematic reviews and Meta‐Analyses (PRISMA) diagram of screening process.

The initial search yielded 162 studies. After removing duplicate records, screening titles, abstracts, and full texts, 13 studies were included in the final review. The primary reasons for exclusion were: studies not specifically addressing hikikomori or pathological social withdrawal; absence of reference to autism or DSM‐5 diagnostic criteria; lack of empirical data; and publication outside of the 2022–2025 date range. A PRISMA flow diagram illustrating the selection process is provided in Figure 1. Data extraction encompassed population characteristics, study design, assessment tools, key findings, and interpretations related to adaptive versus pathological outcomes.

### Data synthesis

A thematic synthesis approach was employed. Findings were organized according to the three research questions:

1. Overlap with autistic traits: social communication, sensory sensitivities, restricted interests, and developmental trajectories.
2. Environmental and societal factors: family dynamics, school pressures, cultural expectations, and systemic influences.
3. Adaptive versus pathological framing: functions of withdrawal, cross‐cultural variations, comorbidities, and potential protective effects.

Ecological systems theory provided a lens for mapping environmental and cultural influences, while a neurodiversity‐informed interpretation of adaptive mechanisms. Findings were tabulated and synthesized narratively, with illustrative examples drawn from case studies and international literature.

## INTEGRATED FINDINGS

### RQ1: Overlap between hikikomori and autistic traits

**RQ1**: *What evidence exists for an overlap between autism spectrum traits and hikikomori behaviors in adolescence and emerging adulthood?*

#### Social communication and interaction

Multiple studies report (see Table 1) that individuals exhibiting hikikomori behaviors demonstrate social communication differences reminiscent of autistic traits. Common patterns include the following:

− Few or no close friendships.^15^
− Avoidance of face‐to‐face communication.^2^
− Withdrawal following negative or overwhelming social experiences.^19^

**Table 1:** Overlap between hikikomori and autistic traits.

Autism spectrum disorder (ASD) criteria (DSM‐5)	Hikikomori observations	Key references	Notes/interpretation
Persistent deficits in social–emotional reciprocity, non‐verbal communication, and relationship skills	Often introverted; communication difficulties; few or no close friends; withdrawal following negative social experiences	[2, 9, 15, 19]	Social communication differences suggest overlap with autism but may be situational or culturally mediated
Restricted and repetitive behaviors and insistence on sameness	Severe social withdrawal; intense engagement in gaming or structured routines	[5, 11]	Behaviors may serve as coping mechanisms rather than core ASD traits
Symptoms present in early development	Some case studies report childhood social/behavioral differences	[10]	Suggests underlying traits may exist before the onset of hikikomori, but often unrecognized
Clinically significant impairment	Loss of employment, school refusal, limited social functioning	[20]	Functional impairment aligns with diagnostic criteria for distress
Presentation not better explained by intellectual disability or developmental delay	Emerges later in adolescence/early adulthood; represents change from baseline	[10]	Indicates hikikomori may be a behavioral response rather than a developmental disorder

*Note*: This table illustrates behavioral parallels between autism diagnostic criteria and observed hikikomori characteristics. It does not imply diagnostic equivalence or shared neurodevelopmental etiology. Similarities are interpreted as indicative of potential overlap and shared vulnerability rather than categorical equivalence.

Case studies often describe retrospective accounts of childhood social difficulties, suggesting that subclinical autistic traits may be present long before the onset of withdrawal.^10^, ^21^ Social anxiety appears to mediate the relationship between autistic traits and withdrawal behaviors, functioning as a protective coping response.

#### Sensory processing and restricted interests

Hikikomori individuals frequently report sensory sensitivities and engagement in highly structured, repetitive activities, such as online gaming or solitary hobbies, which parallels the restricted and repetitive behaviors seen in autism.^9^, ^11^ Withdrawal may serve as a strategy for sensory regulation and cognitive predictability.

#### Developmental trajectories

A key distinction lies in timing: autism typically presents in early childhood, whereas hikikomori emerges in adolescence or early adulthood. Retrospective analyses indicate that early signs of neurodivergence may go unrecognized or concealed by masking, particularly in females, where camouflaging behaviors might delay identification and contribute to later withdrawal.^22^, ^23^, ^24^ Additionally, many studies in this area rely on self‐report measures of autistic traits rather than formal diagnosis, which should be considered when interpreting findings. Thus, hikikomori may represent a later, context‐driven manifestation of pre‐existing autistic traits.

### RQ2: Environmental and societal factors in Japan

**RQ2**: *What environmental and societal factors in Japan might trigger hikikomori behaviors in vulnerable individuals?*

The ecological systems framework^25^ facilitates analysis of the multilevel environmental contributors (see Table 2).

**Table 2:** Multilevel analysis of hikikomori utilizing ecological systems approach.

Ecological level	Example	Key findings	Key references	Relevance to RQ2
Microsystem	Family dynamics, peer relationships	High parental overprotection; cultural value of *amae*; normalization of bullying; inflexible school culture	[2, 10, 15, 26, 27, 28, 29]	Social withdrawal initially reduces conflict about school attendance; autistic young people are more likely to be bullied; rigid environments challenge executive functioning
Mesosystem	Connections between family and school	Relatively low autism diagnosis rates in Japan; school refusal often treated as a family matter	[15, 30, 31, 32, 33]	Parents discouraged from challenging teacher decisions; poor family–school communication limits effective support
Exosystem	Education policies, SEND provision	Limited provision for disabled students in mainstream settings	[34, 35, 36, p. 867, 37]	Inadequate inclusion for SEND students; adds financial and social pressure on families
Macrosystem	Cultural values and beliefs	Emphasis on filial piety and collectivism; concept of “true self” versus external self; high‐context communication culture	[2, 28, 38, 39, 40]	Responsibility placed on family; stigma limits access to support; communication norms create barriers for neurodivergent individuals
Chronosystem	Developmental transitions, COVID‐19	Transitional periods heighten susceptibility to withdrawal; economic changes and technological developments complicate life transitions	[16, 41, 42]	Life transitions and broader socioeconomic shifts increase vulnerability to social withdrawal

#### Microsystem: Immediate environments

Family dynamics and parenting styles: Overprotective or co‐dependent family structures and expectations of eldest sons amplify vulnerability to social withdrawal.^14^, ^43^

School environment: Rigid academic structures, high‐stakes assessments, and limited accommodation for neurodivergent learning styles create environments of stress and exclusion.^27^, ^44^

Bullying and peer relationships: Peer victimization is frequently cited as a trigger for withdrawal, especially among individuals with social communication differences.^15^, ^45^

#### Mesosystem: Interactions between microsystems

Family–school communication: Low autism diagnosis rates and limited school interventions impede coordinated support.^30^, ^31^

Cultural attribution of behavior: Children's behavior is often seen as reflecting parental competence, discouraging help‐seeking and reinforcing withdrawal as a protective response.^2^, ^10^

#### Exosystem: Indirect environmental influences

Education and employment systems: Limited policy support for inclusion in schools and rigid labor markets increase stress for individuals with neurodivergent traits.^37^, ^46^

Medicalization of hikikomori: Pathologizing withdrawal risks ignoring systemic and cultural contributors, misattributing societal failures to individual dysfunction.^46^

#### Macrosystem: Cultural norms and societal values

Social harmony and indirect communication: High‐context communication norms may disadvantage autistic individuals, contributing to miscommunication and social isolation.^38^, ^47^

Stigma and gender expectations: Cultural stigma around mental illness and a strong emphasis on male achievement amplify stress and discourage support‐seeking.^31^, ^48^

#### Chronosystem: Broader transitions

Life transitions: Moving from school to work or higher education introduces new social demands and reduced support, often precipitating withdrawal.^42^

Technological development: Internet use and online gaming provide alternative avenues for social connection but may also reinforce isolation patterns.^41^

### RQ3: Adaptive versus pathological conceptualizations

**RQ3**: *To what extent can hikikomori be conceptualized as an adaptive response?*

#### Pathological framing

Many studies categorize hikikomori as pathological, linking it to psychological distress, depression, anxiety, and functional impairment.^12^ Biological markers and psychiatric comorbidities are also documented.^49^

#### Adaptive functions

Evidence suggests that withdrawal can serve protective functions:

- Sensory regulation: Avoiding overstimulating environments reduces stress for individuals with sensory sensitivities.^12^
- Psychological coping: Withdrawal mitigates social anxiety and provides opportunities for intrapersonal reflection.^13^
- Engagement in preferred activities: Online gaming, hobbies, or structured solitary tasks can facilitate mastery, enjoyment, and alternative social connection.^5^, ^8^

#### Cross‐cultural variations

Interpretations of hikikomori differ across contexts. In Japan and Italy, it is often medicalized, whereas in Oman, it is framed as a culturally reactive idiom of distress.^3^ Economic and social instability contributes to withdrawal globally, supporting an adaptive perspective in response to environmental stressors.^6^, ^11^

#### Costs and benefits

Withdrawal may reduce sensory overload and social anxiety, but prolonged isolation carries risks: depression, functional impairment, and social disconnection. Outcomes likely depend on context, support systems, and individual differences in temperament and neurodivergence.^50^, ^51^ Outcomes are therefore best understood not as fixed categories but as context‐dependent and temporally variable, shared by the function of withdrawal, the availability of support, and the quality of fit between individual needs and environmental demands.

Integrated findings summary (see Table 3):

1. Overlap with autistic traits: Social communication differences, sensory sensitivities, and restricted interests are common features, though developmental timing differs.
2. Environmental triggers: Japanese cultural and institutional pressures amplify withdrawal in vulnerable individuals, though similar mechanisms operate internationally under different systemic stressors.
3. Adaptive potential: Withdrawal can serve protective and preference‐based functions, though risks of psychological and functional impairment remain, highlighting the spectrum of outcomes.

**Table 3:** Cross section of undertaken research design.

Author(s), year	Study type/design	Population/sample	Key findings	Interpretation: adaptive versus pathological	Evidential level
Al‐Sibani et al., 2023^3^	Self‐report survey	Cross‐sectional sample from Oman	High levels of HLID; validated HQ‐25	Pathological and adaptive	Moderate
Brosnan and Gavin, 2023^8^	Online questionnaire	646 individuals aged 16–24	Autistic traits mediated relationship between hikikomori risk and psychological well‐being	Pathological	Moderate
Carpita et al., 2024^11^	Case study analysis	40‐ and 20‐year‐old males	Strong correlation between autistic traits and hikikomori risk	Pathological	Lower
Cerbara et al., 2025^53^	Hierarchical cluster analysis	Mixed sample of 14–19‐year‐olds	Identified three distinct social groups	Pathological and adaptive	Moderate
Dell'Osso et al., 2023^5^	Literature review	Multiple studies	Positive correlations between autism and Internet Gaming Disorder	Pathological and adaptive	Lower
Gavin and Brosnan, 2022^51^	Online survey	826 participants aged 16–24	Identified clear risk factors for hikikomori	Primarily pathological	Moderate
Huang et al., 2024^50^	Longitudinal online survey	Adults aged 30–59, proficient in Japanese	Differentiated “pathological hikikomori” as physical isolation with functional impairment or distress	Pathological with some sociocultural considerations	High
Neoh et al., 2023^6^	Systematic review	Multiple studies	Patterns in contributing factors identified	Pathological with some adaptive elements	High
Nonaka et al., 2025^13^	Hikikomori Functional Assessment Scale	Individuals aged 20–64 in Japan	Hikikomori behavior culturally reactive and age‐related	Recognizes pathological, individual, and social factors	Moderate
Santona et al., 2023^49^	Questionnaires	72 participants (49 males, 23 females), aged 12–33	High levels of psychological distress in sufferers	Pathological	Moderate
Yamada et al., 2023^12^	Interviews, questionnaires, blood tests	39 male autistic participants, with and without hikikomori	Higher depressive symptoms, preference for solitude, social anxiety, and sensory sensitivity in hikikomori cases	Pathological	Moderate
Zhang et al., 2025^4^	Meta‐analysis and systematic review	Multiple studies	Cross‐cultural prevalence similar across East Asian and Western samples	Pathological	High

*Note*: Evidential level reflects study design hierarchy. High = meta‐analyses, systematic reviews, and longitudinal studies. Moderate = cross‐sectional surveys, clinical interview studies, and scale development studies. Lower = narrative reviews and case studies. Conclusions are weighted accordingly throughout the synthesis.

Abbreviations: HLID, Hikikomori‐like idiom of distress; HQ, Hikikomori questionnaie.

## DISCUSSION

This discussion interprets the integrated findings of the study in relation to the three research questions. It moves beyond deficit‐based explanations, combining ecological systems theory with a neurodiversity‐informed perspective to reconceptualize hikikomori as a contextually situated phenomenon rather than a purely pathological condition.

### RQ1: Linking hikikomori to autistic traits

The evidence demonstrates a notable convergence between hikikomori behaviors and autistic traits, particularly in social communication, sensory processing, and desire for predictability. Social withdrawal in hikikomori may reflect underlying neurodevelopmental differences, rather than representing a discrete disorder.

#### Social communication and interaction

Hikikomori individuals often exhibit limited friendships, avoidance of verbal communication, and difficulties with theory of mind, reflecting core features of autism.^21^, ^52^ Social anxiety functions as a mediating factor, whereby repeated perceived social failures lead to withdrawal as a protective coping mechanism. This aligns with the double‐empathy problem, which frames social difficulties as mutual miscommunication between autistic and non‐autistic individuals.^47^

#### Sensory sensitivities and behavioral patterns

Withdrawal into the home can serve as a method of sensory regulation and create predictable environments, echoing restricted and repetitive behaviors observed in autism.^5^, ^9^ Engagement in gaming or structured solitary activities may support cognitive stability and psychological well‐being, suggesting these behaviors can have adaptive utility rather than being purely symptomatic.

#### Developmental considerations

Hikikomori emerges in adolescence or early adulthood, contrasting with the early onset of autism. Retrospective accounts suggest that subtle neurodevelopmental differences may exist from childhood but remain unrecognized, especially in females.^23^, ^24^ This delayed manifestation underscores the importance of early identification and the risks of diagnostic biases.

It is important to acknowledge the considerable heterogeneity within autism as a condition. Autism encompasses a wide spectrum of intellectual functioning, from individuals with significant cognitive disabilities to those with notable intellectual ability. Masking and camouflaging behaviors, whereby autistic individuals suppress or conceal autistic traits to navigate social expectations, are particularly prevalent among females and may account for delayed or missed identification of underlying neurodivergent traits in hikikomori populations.^22^, ^23^ Co‐occurring psychiatric conditions, including anxiety, depression, and OCD, are common in autism and may independently contribute to social withdrawal, complicating the interpretation of behavioral overlap with hikikomori. Furthermore, a distinction should be drawn between formally diagnosed autism and subclinical autistic traits measured through self‐report questions such as the Autism Quotient (AQ). Many studies here reply on trait‐based measures rather than clinical diagnosis, which limits the strength of conclusions regarding neurodevelopmental etiology and introduces potential measurement bias.

#### Synthesis

The findings suggest that hikikomori is not simply undiagnosed autism but may represent a contextually mediated expression of underlying autistic traits. Interventions should recognize these traits while considering the broader environmental factors that shape withdrawal behaviors.

It should be noted that the available evidence, predominantly cross‐sectional and correlational in design, supports behavioral overlap and risk association between autistic traits and hikikomori, but does not establish developmental or etiological continuity. The directionality of this relationship remains unclear, and shared third variables such as anxiety, trauma, or environmental stressors may account for observed associations.

### RQ2: Environmental and societal influences in Japan

The ecological systems framework reveals that hikikomori is shaped by multilevel interactions between individual vulnerabilities and environmental stressors.

#### Microsystem

Immediate environments such as family and school play a critical role. Overprotective parenting, high academic expectations, and bullying exacerbate vulnerability, particularly among eldest sons and neurodivergent individuals.^15^, ^26^ Withdrawal often emerges as a short‐term protective response that reduces immediate conflict and distress.

#### Mesosystem

Interactions between family and school systems influence outcomes. Low autism diagnosis rates and cultural interpretations of behavior as parental failure hinder effective support.^30^, ^31^ Limited communication between microsystems reinforces withdrawal as a coping strategy.

#### Exosystem and macrosystem

Broader structural factors, including rigid educational and labor systems, cultural emphasis on social harmony, indirect communication norms, and stigma around mental health, create an environment where withdrawal can be functionally adaptive.^14^, ^38^

#### Chronosystem

Life transitions, economic shifts, technological developments, and events such as COVID‐19 contribute additional pressures. Technological engagement can both mitigate and reinforce social withdrawal, highlighting the importance of context in determining whether withdrawal is adaptive or detrimental.^41^, ^42^

#### Synthesis

Hikikomori emerges from a dynamic interaction of individual neurodivergent traits in combination with layered ecological factors that reinforce one another over time. For example, rigid educational structures at the exosystem level might interact with family dynamics at the microsystem level, creating compounding pressures that can precipitate withdrawal during key developmental transitions. It is important to differentiate between structural conditions that are specific to Japan and globally observed patterns. Features such as high‐context communication norms, filial piety, and cultural stigma around mental health difficulties are particularly salient in Japan and shape the form that hikikomori behavior takes in that specific context. Internationally, analogous withdrawal behaviors emerge under different systemic conditions, including socioeconomic instability, precarious employment opportunities, and disruption related to the pandemic, which suggests that ecological misattunement rather than cultural specificity is the core mechanism.

Importantly, Japan's institutional landscape is not static. Autism diagnosis rates in Japan have increased substantially over the past decade,^54^ with potential implications for how hikikomori is identified and understood clinically. Similarly, inclusive education policy in Japan has undergone reform, though implementation remains inconsistent, and provision for neurodivergent students in mainstream settings continues to be limited.^35^, ^37^ These contemporary developments are relevant to understanding how hikikomori may evolve as a phenomenon and highlight the need for ongoing policy attention to neurodivergent young people's experiences within educational systems.

### RQ3: Adaptive versus pathological conceptualizations

Before examining the evidence, it is necessary to clarify what is meant by “adaptive” withdrawal in this context. For the purposes of this review, adaptive withdrawal is defined from the perspective of the individual, referring to withdrawal as a behavior that reduces distress, supports sensory regulation, or facilitates engagement in meaningful activities, resulting in preserved or improved subjective well‐being in the short to medium term. This is distinguished from family or societal perspectives, where withdrawal may be viewed as problematic regardless of its function for the individual. This distinction is clinically important: withdrawal that is adaptive for the individual may simultaneously be distressing for the family, or socially unacceptable within a given cultural context.

Temporal considerations are also significant. Withdrawal may serve adaptive functions in the short term, reducing sensory overload, mitigating social anxiety, or providing space for psychological recovery, while also carrying risks of functional impairment, depressed and social disconnection if the withdrawal is prolonged. Nonaka et al.^1^ provide a useful functional typology: intrapersonal‐positive reinforcement and intrapersonal‐negative reinforcement. The former is withdrawal motivated by engagement in enjoyable social activities and associated with preserved well‐being and may represent genuine adaptive preference. Intrapersonal negative reinforcement is motivated by avoidance of social situations, and social negative reinforcement is strongly associated with psychiatric symptoms and reduced quality of life. This framework provides a clinically meaningful basis for distinguishing adaptive from pathological withdrawal that goes beyond simple behavioral description.

The findings indicate that hikikomori exists on a spectrum between pathology and adaptation. While many studies highlight functional impairment, psychological distress, and comorbidities,^12^ others recognize adaptive functions such as:

- *Intrapersonal coping*: Withdrawal can reduce anxiety, regulate sensory input, and enable self‐directed engagement in enjoyable activities.^13^
- *Social‐negative reinforcement*: Avoiding overstimulating social environments may preserve psychological well‐being.
- *Alternative social connection*: Online gaming or digital communities offer controlled, low‐risk social interaction for neurodivergent individuals.^5^, ^8^

Cross‐cultural evidence demonstrates that the framing of hikikomori is context‐dependent. In Japan and Italy, it is often medicalized, whereas in Oman, it is interpreted as a culturally reactive response to environmental stress.^3^ Outcomes depend on individual differences, available support, and environmental fit, emphasizing the need for personalized approaches.

*Implications for support*: Interventions should:

1. Recognize diverse social needs across neurotypes.
2. Facilitate access to meaningful activities during withdrawal.
3. Address systemic barriers rather than focusing exclusively on individual change.
4. Use technology as an engagement tool rather than viewing it uniformly as a risk factor.
5. Evaluate withdrawal functionally, distinguishing between distress‐driven and preference‐based behaviors.

Reconceptualizing hikikomori through a neurodiversity‐informed lens allows for nuanced, context‐sensitive interpretations that balance potential adaptive value with genuine psychological needs.

### Theoretical and practical implications

*Neurodiversity‐informed framework*: This study supports interpreting hikikomori as a functional response in certain contexts, challenging traditional deficit‐focused psychiatric models. It emphasizes the importance of recognizing individual differences, developmental trajectories, and systemic pressures in conceptualizing social withdrawal.

*Ecological systems perspective*: The multilevel analysis highlights the interaction between individual traits and systemic factors. Interventions should target multiple levels, including family, school, policy, and cultural practices, rather than focusing solely on individual behavior.

*Policy and practice*: Flexible educational and employment pathways, inclusive support structures, and culturally sensitive mental health services can mitigate withdrawal while respecting autonomy. Early identification of neurodivergent traits, particularly among females, is critical to prevent long‐term isolation.

*Future research directions*:

− Longitudinal studies tracking outcomes of different withdrawal patterns in autistic individuals.
− First‐person qualitative research exploring lived experiences of hikikomori.
− Ecological studies examining systemic factors that facilitate positive adaptation.
− Development of assessment tools distinguishing between distress‐driven and preference‐based withdrawal.
− Investigation of online social connections as potential adaptive strategies.

## CONCLUSION

This work critically examines the intersection between neurodivergent traits, particularly autism, and hikikomori. The synthesis of three research questions provides the following key clear insights:

*Hikikomori as a potential neurodivergent expression*: There is a consistent pattern of behavioral overlap between hikikomori and autistic traits across the reviewed literature. While this overlap does not establish causal or etiological continuity, it suggests that autistic traits may represent a significant vulnerability factor under specific ecological conditions.

*Environmental stressors*: Social, cultural, and institutional pressures, especially in Japan, interact with individual vulnerabilities to produce withdrawal. Educational rigidity, familial expectations, bullying, indirect communication norms, and stigma contribute to the emergence and maintenance of hikikomori behaviors. Internationally, similar mechanisms operate under different socioeconomic conditions, highlighting the universal relevance of ecological factors.

*Adaptive and pathological dimensions*: Hikikomori exists on a continuum from adaptive coping to pathological dysfunction. Withdrawal may serve protective, sensory‐regulating, and preference‐based functions, but prolonged isolation can contribute to depression, functional impairment, and social disconnection. Understanding withdrawal in context allows for balanced, personalized support approaches.

*Implications for practice and policy*:

− Mental health professionals should adopt neurodiversity‐informed approaches when working with socially withdrawn individuals.
− Educational and employment systems must implement flexible, inclusive practices accommodating diverse neurotypes.
− Technology can be a valuable tool for maintaining social connection and engagement during periods of withdrawal.
− Policies should address systemic barriers and facilitate cross‐sector collaboration to support isolated individuals while respecting autonomy.

*Final reflections*: Hikikomori exemplifies the need to reconceptualize mental health, neurodiversity, and social functioning. Withdrawal may reflect rational adaptation to environmental misattunement rather than inherent dysfunction. This challenges deficit‐based frameworks and invites consideration of systemic change to support the full spectrum of human neurodiversity. Future research and policy must focus not only on reintegration into mainstream society but also on cultivating environments that accommodate diverse developmental and social needs.

## AUTHOR CONTRIBUTIONS

Emily Ravenhill and William Farr conceived the study. Emily Ravenhill conducted the experiments and analyzed the data. William Farr supervised the project. Emily Ravenhill wrote the original draft, and both authors reviewed and edited the manuscript.

## CONFLICT OF INTEREST STATEMENT

The authors declare no conflicts of interest.

## ETHICS APPROVAL STATEMENT

Ethical approval was sought and obtained from the Faculty Board, Faculty of Education, University of Cambridge, March 2025.

## PATIENT CONSENT STATEMENT

Patient consent for publication was not required as all data were fully anonymized and no identifiable information is included.

## CLINICAL TRIAL REGISTRATION

N/A.

## Data Availability

The data that support the findings of this study are available from the corresponding author upon reasonable request.
